# Studying executive functions in senior preschoolers

**DOI:** 10.1002/pchj.310

**Published:** 2019-08-02

**Authors:** Alexander Veraksa, Olga Almazova, Daria Bukhalenkova

**Affiliations:** ^1^ Faculty of Psychology Lomonosov Moscow State University Moscow Russian Federation

**Keywords:** cognitive flexibility, executive functions (EF), inhibition, preschool age, working memory

## Abstract

This study analyzed connections between different components of executive functions (EF; inhibition, working memory, cognitive flexibility) among 1,075 preschool children in Moscow. The results suggested greater heterochronicity in different EF component levels for girls compared with boys. Factor analysis showed the best fit for a three‐factor model.

Most researchers consider the development of executive functions (EF) to be one of the most important achievements in senior preschoolers and to serve as a predictor of children's successful performance and adaptation to school (Nisskaya, 2018; Willoughby, Kupersmidt, & Voegler–Lee, 2012).

According to A. Miyake's model, the neuropsychological basis for mastering one's own behavior consists of a group of cognitive skills that provide targeted problem solving and adaptive behavior in new situations; these skills have come to be generally known as executive functions. They help to monitor and control thought and activities by shifting these processes toward the task‐related stimulus despite the presence of secondary tasks and interference (Miyake et al., 2000). EF are divided into the following main components: (1) working memory, both visual and verbal, (2) cognitive flexibility, which is related to the ability to switch from one rule to another, and (3) inhibitory control, which presupposes the inhibition of the dominant response in favor of what is required to perform the task.

Though related, these components can also be considered as being independent and separate from each other, which is why this model has come to be called “unity‐with‐diversity (Miyake et al., 2000).” Most studies carried out with 3–5‐year‐old preschoolers support a unitary model, but some of these studies measure only two of the EF components (except working memory or flexibility). Other studies suggest a two‐factor model of EF in which inhibition and flexibility are combined into one factor, and working memory into another; or a model in which inhibition is allocated as a separate factor, and working memory is combined with cognitive flexibility (Monette, Bigras, & Lafrenière, 2015; Usai, Viterbori, Traverso, & De Franchis, 2013).

The study involved 1,075 preschoolers aged 5–6 years (m = 5.5) in Moscow. Of these, 51.7% were boys and 48.3% were girls. The parents of the preschoolers gave their written informed consent for their children's participation in the study. The procedure was approved by the institutional review board of the Faculty of Psychology of Lomonosov Moscow State University.

To assess EF development, we employed a set of four methods widely used in psychological practice (Almazova, Bukhalenkova, Veraksa, & Martinenko, 2017). Most of the methods consisted of NEPSY‐II subtests (Korkman, Kirk, & Kemp, 2007).

The working memory level was measured by using the Sentences Repetition subtest for verbal working memory (17 sentences of increasing length, syntaxes, and semantic complexity: total score max = 34 points) and the Memory for Designs subtest from NEPSY‐II for visual and spatial working memory (4 trials in which child ought to select the appropriate designs and place them on a grid in the same location as previously shown: total score max = 120 points).

Cognitive flexibility and inhibition were assessed using the Dimensional Change Card Sort test (DCCS; Zelazo, 2006) and Inhibition subtest (NEPSY‐II). The Inhibition subtest consisted of two tasks: (1) Naming: The child was asked to name the shape of figures (black and white squares and circles) on the page as quickly as possible); and (2) Inhibition: The child was required to name figures that were the opposite of those actually pictured (say “circle” instead of “square” and vice versa). The number of errors (both corrected by a child and not corrected) and the completion time to both tasks were recorded.

When identifying the relations between different components of the EF (Pearson's correlation coefficient) for the entire sample, the following pairs were found to be more closely interrelated: (1) the level of verbal working memory and cognitive flexibility on the DCCS (r = .279, *p* < .001); and (2) the level of visual working memory and inhibition (r between .153 and .207, *p* < .001). At the same time, there is no connection in the assessments for such EF components as cognitive flexibility and inhibition.

Regarding the level of different EF components in boys and girls, the following differences were obtained (*t*‐test for pairs of independent samples). Girls performed significantly better than boys on tasks pertaining to working memory (verbal: *t* = −2.990, *p* = .003; girls: *M* = 19.08, *SD* = 4.56; boys: *M* = 18.23, *SD* = 4.66) as well as detail memorization for visual memory (*t* = −2.293, *p* = 0.022; girls: *M* = 38.58, *SD* = 5.48; boys: *M* = 37.75, *SD* = 5.89) and cognitive flexibility (*t* = −5.148, *p* < .001; girls: *M* = 19.34, *SD* = 2.82; boys: *M* = 18.34, *SD* = 3.44) . Girls also made fewer mistakes than boys on inhibition tasks (Uncorrected Errors: *t* = 3.075, *p* = .002; girls: *M* = 2.56, *SD* = 5.59; boys: *M* = 3.74, *SD* = 6.64; Corrected Errors: *t* = 2.207, *p* = .028; girls: *M* = 1.99, *SD* = 1.91; boys: *M* = 2.26, *SD* = 2.15).

Having separately examined the connections between different EF components for boys and girls (Pearson's correlation coefficient), we identified the following differences:

1. Unlike girls, boys were found to show a significant correlation between the level of their verbal and visual working memory (boys: r = .217, *p* < .001; girls: r = .068, *p* = .142).
2. In boys, the developmental level of verbal working memory was related to the time spent doing the inhibition task, whereas in girls this level was unrelated (boys: r = −.157, *p* < .001; girls: r = −.057, *p* = .210).
3. In boys, the developmental level of visual working memory was associated with cognitive flexibility, whereas in girls it was unrelated (boys: r = .223, *p* < .001; girls: r = .040, *p* = .387).

Thus, different EF components are more closely related to each other in boys than in girls, which suggests a greater heterochronicity in the level of different EF components in girls.

Confirmatory factor analysis results (the method of principal components, rotation varimax) show three factors describing 53% of the variance. The first factor includes all parameters related to the measurement of visual working memory (factor loads from 0.775 to 0.986). The second factor includes parameters of verbal working memory (factor loads = 0.305) and cognitive flexibility (factor loads from 0.935 to 0.937). The third factor includes the number of mistakes and the completion time on the inhibition task (factor loads from 0.319 to 0.730).

Thus, the study revealed several significant links between EF components in children aged 5–6 years.

First, the data showed a higher level of working memory and inhibition in girls compared with boys, as well as gender differences in the relationship of the EF components. The revealed gender differences are consistent with a number of recent studies (e.g., Cadavid‐Ruiz, Río, Egido, & Galindo‐Villadrón, 2016; Montroy, Bowles, & Skibbe, 2016); however, many studies show no significant difference between girls and boys at this age (e.g., Monette et al., 2015). These results can be explained by differences in speech development (Cadavid‐Ruiz et al., 2016) between boys and girls or by the specifics of boys’ playing activity with peers in comparison with girls’ (Montroy et al., 2016).

Second, we developed a three‐factor model of EF in the senior preschool age range in contrast to previous studies (Monette et al., 2015; Usai et al., 2013). The differences that we discovered could be due to use of raw rather than standardized scores. We did not compute the total score for the Inhibition subtest, but we have controlled the nonverbal intelligence level in the research.

Thus, the resulting variables are convenient and informative in assessing EF levels as predictors of cognitive and emotional development in senior preschoolers.

## References

[pchj310-bib-0001] Almazova, O. V. , Bukhalenkova, D. A. , Veraksa, A. N. , & Martinenko, M. N. (2017). Diagnosis of the level of development of executive functions European Proceedings of Social & Behavioural Sciences (EpSBS), 33, 406–416. 10.15405/epsbs.2017.12.43

[pchj310-bib-0002] Cadavid‐Ruiz, N. , Río, P. , Egido, J. , & Galindo‐Villadrón, P. (2016). Age related changes in the executive function of Colombian children. Universitas Psychologica, 15(5), 1–10. 10.11144/Javeriana.upsy15-5.arce

[pchj310-bib-0003] Korkman, M. , Kirk, U. , & Kemp, S. L. (2007). NEPSY II. Administrative manual. San Antonio, TX: Psychological Corporation.

[pchj310-bib-0004] Miyake, A. , Friedman, N. P. , Emerson, M. J. , Witzki, A. H. , Howerter, A. , & Wager, T. (2000). The unity and diversity of executive functions and their contributions to complex “frontal lobe” tasks: A latent variable analysis. Cognitive Psychology, 41, 49–100. 10.1006/cogp.1999.0734 10945922

[pchj310-bib-0005] Monette, S. , Bigras, M. , & Lafrenière, M. (2015). Structure of executive functions in typically developing kindergarteners. Journal of Experimental Child Psychology, 140, 120–139. 10.1016/j.jecp.2015.07.005 26241760

[pchj310-bib-0006] Montroy, J. J. , Bowles, R. P. , & Skibbe, L. E. (2016). The effect of peers’ self‐regulation on preschooler's self‐regulation and literacy growth. Journal of Applied Developmental Psychology, 46, 73–83. 10.1016/j.appdev.2016.09.001

[pchj310-bib-0007] Nisskaya, A. K. (2018). School readiness outcomes of different preschool educational approaches. Psychology in Russia. State of the Art, 11(1), 43–60. 10.11621/pir.2018.0104

[pchj310-bib-0008] Usai, M. C. , Viterbori, P. , Traverso, L. , & De Franchis, V. (2013). Latent structure of executive function in five‐ and six‐year‐old children: A longitudinal study. European Journal of Developmental Psychology, 11, 447–462. 10.1080/17405629.2013.840578

[pchj310-bib-0009] Willoughby, M. T. , Kupersmidt, J. B. , & Voegler–Lee, M. E. (2012). Is preschool executive function causally related to academic achievement? Child Neuropsychology, 18(1), 79–91. 10.1080/09297049.2011.578572 21707258 PMC3417807

[pchj310-bib-0010] Zelazo, P. D. (2006). The Dimensional Change Card Sort (DCCS): A method of assessing executive function in children. Nature Protocols, 1, 297–301. 10.1038/nprot.2006.46 17406248

